# Effect of Clothing Fabric on 20-km Cycling Performance in Endurance Athletes

**DOI:** 10.3389/fspor.2021.735923

**Published:** 2022-01-05

**Authors:** Jared Ferguson, Amir Hadid, Yoram Epstein, Dennis Jensen

**Affiliations:** ^1^Clinical Exercise & Respiratory Physiology Laboratory, Department of Kinesiology & Physical Education, McGill University, Montréal, QC, Canada; ^2^McGill's Sports Science Institute, McGill University, Montréal, QC, Canada; ^3^Heller Institute of Medical Research, Sheba Medical Center, Tel Hashomer, Sackler Faculty of Medicine, Tel Aviv University, Tel Aviv, Israel; ^4^Sylvan Adams Sports Institute, Tel-Aviv University, Tel-Aviv, Israel

**Keywords:** 20-km cycling time, exercise, thermoregulation, natural fabrics, synthetic fabrics

## Abstract

**Purpose:** Examine the effect of synthetic fabrics (SYN, 60% polyester: 40% nylon) vs. 100% cotton fabric (CTN) on the 20-km cycling time trial (20 kmCTT) performance of competitive cyclists and triathletes.

**Methods:** In this randomized controlled crossover study, 15 adults (5 women) aged 29.6 ± 2.7 years (mean ± SE) with a peak rate of O_2_ consumption of 60.0 ± 2.0 ml/kg/min completed a 20 kmCTT under ambient laboratory conditions (24.3 ± 0.7°C and 17 ± 7% relative humidity) with a simulated wind of ~3 m/s while wearing SYN or CTN clothing ensembles. Both ensembles were of snowflake mesh bi-layer construction and consisted of a loose-fitting long-sleeved shirt with full-length trousers.

**Results:** Participants maintained a significantly (*p* < 0.05) higher cycling speed and power output over the last 6-km of the 20 kmCTT while wearing the SYN vs. CTN ensemble (e.g., by 0.98 km/h and 18.4 watts at the 20-km mark). Consequently, 20 kmCTT duration was significantly reduced by 15.7 ± 6.8 sec or 0.8 ± 0.3% during SYN vs. CTN trials (*p* < 0.05). Improved 20 kmCTT performance with SYN vs. CTN clothing could not be explained by concurrent differences in esophageal temperature, sweat rate, ratings of perceived exertion and/or cardiometabolic responses to exercise. However, it was accompanied by significantly lower mean skin temperatures (~1°C) and more favorable ratings of perceived clothing comfort and thermal sensation during exercise.

**Conclusion:** Under the experimental conditions of the current study, athletic clothing made of synthetic fabrics significantly improved the 20 kmCTT performance of endurance-trained athletes by optimizing selected thermoregulatory and perceptual responses to exercise.

## Introduction

In humans, body temperature is regulated under conditions of physical and/or environmental heat stress through the control of net heat loss *via* evaporative and dry heat exchange (Steudel-Numbers, 2003; Kenny and Jay, 2013). Exercise represents a challenge to thermoregulation, with an inverse relationship existing between exercise performance and both skin and core temperature (Rowell et al., 1966; Gonzalez-Alonso et al., 1999; Sawka et al., 2012). Evaporation of sweat from the skin surface accounts for ~55% of heat loss during exercise in human subjects (Plowman and Smith, 2011), and when the ambient temperature is equal to skin temperature, sweat evaporation accounts for almost all heat loss (Sawka and Wenger, 1988). Thus, any intrinsic and/or extrinsic factor capable of facilitating evaporative heat loss and preventing excessive increases in skin temperature has the potential to improve exercise performance. This is of special importance for endurance-trained athletes who, by virtue of their capacity to exercise at relatively high power outputs and metabolic rates, are able to achieve and sustain high net rates of body heat production, i.e., endogenic thermal strain.

The boundary layer on the exterior of the body created by clothing represents one of the most significant barriers to evaporative heat loss with attendant increased rates of heat storage and potential for impaired performance. With advancements in textile technologies, clothing made of synthetic fabrics can be designed to allow for lower water vapor and thermal resistance, in addition to superior moisture management capacity compared to traditional natural fabrics such as cotton or wool (Wang et al., 2014, 2016). Therefore, these synthetic fabrics have the potential to enhance exercise performance by optimizing thermoregulatory and perceptual responses to exercise (Jiao and Yao, 2017). The results from earlier studies concerning the effect of clothing made of synthetic vs. natural fabrics on exercise performance and perceptual responses are inconsistent and inconclusive, with most but not all studies reporting no demonstrable effect (Gavin et al., 2001; Gavin, 2003; Brazaitis et al., 2010; Davis and Bishop, 2013; Sperlich et al., 2013; De Sousa et al., 2014; Abdallah et al., 2015; Corbett et al., 2015), or an effect that was observed among females only (Hooper et al., 2015). A recent study focused on synthetic fabrics with body-mapping design reported improved exercise performance (shorter run time) compared to common synthetic running clothing ensembles (Jiao and Yao, 2017).

Thus, the question remains: *Does clothing made of synthetic fabric(s) vs. natural fabric(s) enhance exercise performance by optimizing thermoregulatory and perceptual responses to exercise?*

The purpose of this study was to address this question by examining the effect of clothing made of engineered synthetic fabrics (SYN) vs. 100% cotton fabric (CTN) on 20-km cycling time trial (20kmCTT) performance in endurance-trained athletes under ambient laboratory conditions with a simulated wind of ~3 m/s. We hypothesized that exercise performance would be significantly improved under SYN vs. CTN clothing conditions, as evidenced by a decrease in 20kmCTT duration. We further hypothesized that SYN-induced enhancement of 20kmCTT performance would be associated with concomitant improvements in heat dissipation, cardiometabolic and perceptual responses to exercise.

## Methods

### Participants

Ten men and 5 women, all active members of a competitive cycling and/or triathlon team/club, completed the study. Their general characteristics are summarized in Table 1. Inclusion criteria were: healthy, non-smoking, non-obese men and women aged ≥18 years with normal spirometry (forced expiratory volume in 1-sec [FEV_1_] ≥80% predicted and a FEV_1_-to-forced vital capacity ratio >70%) and a symptom-limited peak rate of O_2_ consumption (VO_2peak_) ≥60 ml/kg/min and/or ≥125% of the predicted normal value on incremental cardiopulmonary cycle exercise testing (CPET). Exclusion criteria were: taking doctor-prescribed medications other than oral contraceptives and/or had a history of gastrointestinal, cardiovascular, respiratory, kidney, liver, musculoskeletal, endocrine, neuromuscular and/or metabolic disease/disorder. Pregnancy was ruled out by standard (urine) pregnancy test prior to study enrolment.

**Table 1:** Participant characteristics.

**Parameter**	
Sex, male:female	10:5
Age, yrs	29.6 ± 2.7 [20–63]
Body height, cm	175.4 ± 2.5 [161–194]
Body mass, kg	71.3 ± 2.3 [56.3–83.2]
BMI, kg/m^2^	23.2 ± 0.6 [18.1–27.8]
FEV_1_, % predicted	101 ± 2 [93–114]
FEV_1_/FVC, %	78 ± 2 [70–89]
Peak VO_2_, ml/kg/min	60.0 ± 2.0 [47.2–76.8]
Peak VO_2_, % predicted	150 ± 7 [115–191]
Peak power output, watts	393 ± 20 [275–500]
Peak power output, % predicted	186 ± 6 [147–222]
***Self–reported training regime***	
Total endurance training, min/week	518 ± 44 [270–780]
Cycling, training sessions/week	3.7 ± 0.5 [2–7]
Cycling, min/training session	83 ± 8 [30–150]
Running, training sessions/week	2.0 ± 0.4 [0–5]
Running, min/training session	48 ± 9 [0–90]
Swimming, training sessions/week	1.4 ± 0.3 [0–3]
Swimming, min/training session	47 ± 11 [0–120]
Resistance training, training sessions/week	0.9 ± 0.2 [0–2]
Resistance training, min/training session	27 ± 8 [0–90]

*Values are means ± SE [range]. BMI, body mass index; FEV_1_, forced expiratory volume in 1-sec; FVC, forced vital capacity; VO_2_, rate of oxygen uptake*.

We instructed the participants to avoid strenuous exercise, alcohol, and xanthine-containing foods/drinks on each test day. Each visit was separated by ≥48 h and conducted at the same time of day (± 1 h) for each participant. The study protocol and consent form were approved by the Institutional Review Board of the Faculty of Medicine at McGill University (A01-M12-15BB) and conformed to the ethical standards set by the *Declaration of Helsinki*. Each participant signed an informed consent form.

### Study Design

In this randomized controlled crossover study participants visited the *Clinical Exercise and Respiratory Physiology Laboratory* at McGill University on 3 separate occasions over a period of ≤ 21 days. *Visit 1* included screening for eligibility; completion of the Physical Activity Readiness Questionnaire (http://eparmedx.com/wp-content/uploads/2021/01/ParQ-Plus-Jan-2021-Image.pdf) to rule out any medical contraindications to CPET; documentation of training history *via* self-report; spirometry; an incremental CPET to determine VO_2peak_ and peak power output (PPO); and a 5-km cycling time trial (5kmCTT) with faux measurement of esophageal temperature (*T*_eso_) for familiarization purposes. *Visits 2 and 3* included a 20kmCTT with measurement of *T*_eso_ and skin temperature (*T*_skin_) while wearing one of two randomly assigned clothing ensembles: CTN or SYN.

Prior to the 20kmCTT at *visits 2 and 3*, we fitted participants with *T*_eso_ and *T*_skin_ thermistors, and skin surface electrodes for heart rate monitoring by 12-lead ECG. Participants then laid supine for ≥15-min at rest (without simulated wind) while baseline measures of *T*_eso_ and *T*_skin_ were collected. The 20kmCTT commenced once baseline measures of *T*_eso_ varied by ≤ 0.1°C for ≥5-min. Immediately before and after each 20kmCTT, we recorded the participant's nude and dry body mass to the nearest 2 g (KCC 150, Mettler-Toledo Inc., Mississauga, ON, Canada); the mass of the clothing ensemble to the nearest 0.01 g (ALD 4102; Dini Argeo, Modena, Italy); and the mass of the mouthpiece and spit trap to the nearest 0.01 g (ALD 4102). We adjusted pre-exercise body mass for the volume of water consumed during insertion of the *T*_eso_ thermistor, where the water was kept at ambient temperature. After the ingestion of the esophageal thermistor- drinking was not permitted. We controlled temperature and relative humidity under all experimental sessions. They were monitored using Barometric Pressure/Humidity/Temperature Datalogger (Model SD700, EXTECH Instruments, Canada) and were maintained at 24.3 ± 0.7°C and 17 ± 7%.

### Clothing Ensembles

**Figure 1:**
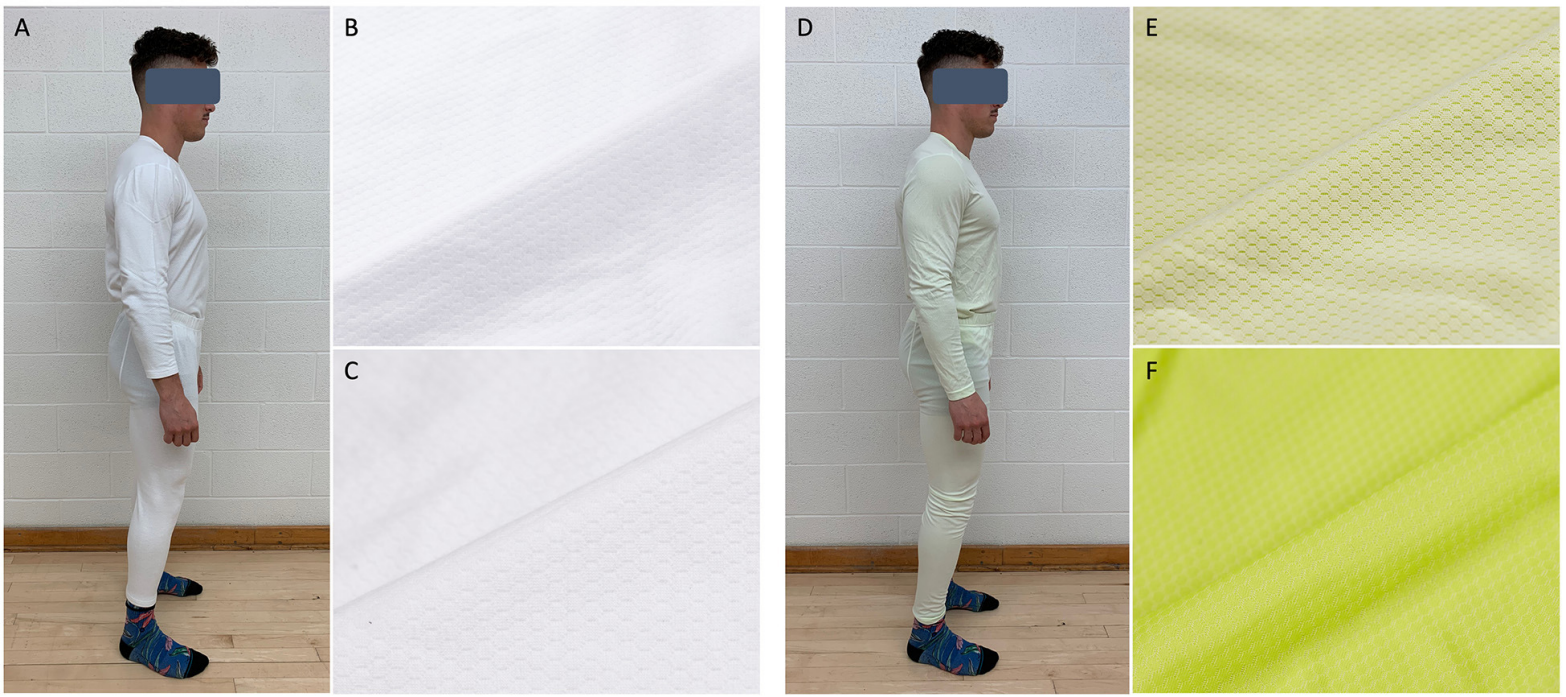
Clothing ensembles. **(A)** 100% cotton fabric (CTN) clothing ensemble (shirt and pants). **(B)** CTN macroscopic structure (face side). **(C)** CTN macroscopic structure (skin side). **(D)** nylon-polyester synthetic fabric (SYN) clothing enemble. **(E)** SYN macroscopic structure (face side) includes channeled polyester to help spread moisture across the surface, designed to promote quicker evaporation. **(F)** SYN macroscopic structure (skin side) includes a flat channel nylon with elements extruded in the yarn to promote cooling effect.

Both SYN and CTN clothing ensembles were of snowflake mesh bi-layer construction and consisted of a loose-fitting long-sleeved shirt tucked into the waistband of a full-length trouser (Figures 1A–F) (Lamour Hosiery Inc., Montreal, QC, Canada). While most commercial sportswear clothing is made of polyester or nylon, our study utilized a polyester-nylon combination. Its construction and yarns were selected following design optimization cycles aimed at maximizing thermal effusively, moisture management and breathability (data not shown here proprietary information of Lamour Inc).

Thermal resistance and water vapor resistance under steady state conditions were evaluated by a certified lab (CTT Group, Saint-Hyacinthe, QC, Canada) using a sweating guarded hotplate system according to ISO 11092:2014. Thermal resistance was measured under climatic room conditions of 20°C and 65% RH. Water vapor resistance was measured under climatic room conditions of 35°C and 40% RH (Table 2). Air permeability was evaluated by a certified lab (SGS North America, Fairfield, NJ, USA) according to ASTM D373-04 2016. Stretch and recovery tests were conducted by Lamour Inc., Montreal, Canada. Stretchability was assessed according to ASTM D2594 (10 lbs) and Recovery according to ASTM D2594 (65% Width-35% Length) and reported for 60 sec and 1 h. All SYN and CTN clothing ensembles were machine-washed and air-dried prior to use in experimental testing. We instructed the participants to wear the same pair of their own cycling shoes and undergarments (i.e., underwear, socks and bra) at *visits 2 and 3*; and to machine-wash and air-dry their undergarments between *visits 2 and 3*.

**Table 2:** Clothing ensembles characteristics.

**Clothing ensemble**	**Composition**	**Weight [g/m^**2**^]**	**Thickness [mm]**	**Thermal resistance (CLO)**	Vapor resistance (Ret) [m^2^*Pa/W]	**Air permeability [cm^**3**^/s/cm^**2**^]**	**Stretch [%]**		**Recovery [%]**			
									**60 sec**		**1 h**	
							**W**	**L**	**W**	**L**	**W**	**L**
Synthetic (SYN)	60% polyester: 75 denier/ 72 filaments 40% nylon: 50 denier/ 24 filaments	130	0.58	0.08 ± 0.00	1.75 ± 0.46	192.3 ± 5.1	90	40	6	2	3	2
Cotton (CTN)	100% cotton: Yarn size, 50 singles	160	0.78	0.22 ± 0.02	5.07 ± 0.29	73.2 ± 1.4	125	50	14	8	9	4

*Values are means ± SD*.

To minimize potential effects of different air gaps due to sizing variation between the SYN and CTN, both clothing ensembles were constructed by the same manufacturer using similar sizing, cut and fit, while the same size pant and shirt was worn for each participant between the 2 conditions.

### Pulmonary Function Testing

We performed spirometry with participants seated using automated equipment (SensorMedics Vs229d; Carefusion, Yorba Linda, CA, USA).

### Cycle Exercise Testing

We conducted exercise tests on Velotron Pro cycle ergometer in combination with Version 1.6 of the Velotron Coaching Software (RacerMate Inc., Seattle, WA, USA) and a Vmax Encore® metabolic cart (Carefusion). Cardiac, metabolic and ventilatory parameters were collected breath-by-breath at rest and during exercise while participants breathed through a rubber mouthpiece and low-resistance flow transducer with nasal passages occluded by a nose clip. Participants remained seated for the duration of each exercise test. We adjusted the position of both the cycle ergometer seat and handlebars to each participant's preference prior to *visit 1* and kept it constant for all subsequent visits.

Incremental exercise (without simulated wind) consisted of a steady-state rest period of ≥6-min, followed by 50 watt/min increases in power output (starting at 50 watts) for men and 25 watt/min increases in power output (starting at 25 watts) for women: PPO was defined as the highest power output the participant was able to sustain for ≥30-sec, while VO_2peak_ was taken as the average of the last 30-sec of loaded pedaling.

Cycling time trials included a pre-exercise rest period of ≥3-min while seated on the cycle ergometer without simulated wind, followed by 1-km of cycling at a power output corresponding to 20% of PPO (warm-up) without simulated wind, and then either a 5kmCTT (*visit 1*) or 20kmCTT (*visits 2 and 3*). To promote convective heat dissipation, “we applied a simulated wind of ~3 m/s for the duration of each cycling time trial using two vertically stacked 20” fans (Comfort Zone CZHV20B, Global Industrial Canada, Richmond Hill, ON, Canada) positioned 1.25 m in front of the cycle ergometer. Air speed was measured using digital anemometer (model 06-664-28, FB61321, 255TX, Control Company, Friendswood, TX, USA). We instructed the participants to complete each time trial as fast as possible by maintaining the highest cycling speed and power output as possible. Except for evaluating perceptual responses, we did not provide verbal feedback, encouragement and/or instruction to the participants. During each cycling time trial, participants received real-time visual feedback on their speed, pedal cadence, distance and gear, while being blinded to test duration.

### Skin and Esophageal Temperature Measurement

We took continuous recordings of: (a) *T*_skin_ from the right upper arm (*T*_arm_), the chest at the right pectoralis (*T*_chest_), the middle of the right vastus lateralis (*T*_thigh_) and the back at the right latissimus dorsi (*T*_back_) rest and during exercise using rapid response skin thermistors (SST-1; Physitemp Instruments Inc., Clinton, NJ, USA) secured to the skin with adhesive dressings (Tegaderm; 3M Health Care, Neuss, Germany); and (b) *T*_eso_ – an index of core body temperature – at rest and during exercise using a rapid response esophageal thermistor (ESO-1; Physitemp Instruments Inc., Accuracy of 0.1°C). After ‘numbing' of the nasal and pharyngeal passages with a 2% endotracheal lidocaine spray (Lidodan^TM^; Odan Laboratories Ltd., Montreal, QC, Canada), we inserted the *T*_eso_ thermistor through the nose and positioned it in the esophagus at a depth of one quarter of the participant's standing height, which is equivalent to the location of the lower third of the esophagus. All temperatures were sampled at 60 Hz and digitized using the Thermes USB temperature data acquisition system in conjunction with DASYLab Basic Software (Physitemp Instruments Inc.). Before each test, the thermistors and Thermes USB system were calibrated as per manufacturer's instructions.

### Analysis of Exercise End-Points

We averaged temperature measurements over the last 3-min of the baseline (supine) rest period and subsequently linked them with cardiac, metabolic and ventilatory parameters averaged over the last 30-sec of the pre-exercise rest period while seated. Temperature, cardiac, metabolic, ventilatory and cycle performance variables were averaged over the last 250-m of every 2-km interval of the 20kmCTT.

### Calculations

We calculated whole body mean skin temperature ($\bar{T}sk$, in °C) using the following equation modified from Park and Palmes (1947): (0.115 x *T*_arm_) + (0.311 x *T*_thigh_) + (0.287 x *T*_chest_) + (0.287 x *T*_back_). Sweat rate (in L/h) was calculated as: ΔBM/*t*, (Gavin et al., 2001) where ΔBM is the exercise-induced change in participant's body mass, and *t* is the time needed to complete the 20kmCTT.

### Perceptual Responses and Debriefing

At rest prior to exercise and within the last 250-m of every 2-km interval during each 20kmCTT, participants provided ratings of: perceived exertion using Borg's 0-10 category ratio scale (Borg, 1982); and clothing comfort, thermal sensation, and skin wettedness (Table 3). Upon completion of all procedures at *visit 3*, participants responded to debriefing questions (Table 3).

**Table 3:** Perceptual and debriefing responses.

**Clothing comfort**								
**+3**	**+2**	**+1**	**0**	**−1**	**−2**	**−3**		
very comfortable	comfortable	slightly comfortable	neutral	slightly uncomfortable	uncomfortable	very uncomfortable		
**Thermal sensation**								
**+** **4**	**+3**	**+2**	**+1**	**0**	**−1**	**−2**	**−3**	**−4**
very cold	cold	cool	slightly cool	neutral	slightly warm	warm	hot	very hot
**Skin wettedness**								
**+** **3**	**+2**	**+1**	**0**	**−1**	**−2**	**−3**		
too dry	dry	slightly dry	neutral	slight wet	wet	too wet		
**Debriefing questions**								
1. During which visit did you feel you wore the garment made of synthetic fabrics?								
2. Overall, during which visit did you feel that you performed the 20kmCTT best?								
a. Did you find the clothing garment you wore to be a factor that contributed to your perception of ‘best' overall performance during the visit you selected in Question 2								
b. If you answered ‘yes' to Question 2a, can you please explain what characteristics of the clothing garment, if any, contributed to your perception of ‘best' overall performance								
3. Overall, during which visit did you experience the least amount of thermal strain during the 20kmCTT?								
4. Overall, during which visit did you think the clothing garment provided the best ‘cooling' effect during the 20kmCTT?								
5. Overall, the clothing ensemble worn during which visit is best suited for your training and/or competition needs?								
6. As a consumer of athletic clothing, can you please identify the visit during which you wore the clothing ensemble that you would most like to purchase for your training and/or competition needs?								

### Sample Size Estimation and Statistical Analyses

Our primary end-point was the time needed to complete the 20kmCTT. Using a two-tailed paired subject formula (Faul et al., 2009) with α = 0.05, β = 0.90 and an expected effect size of 1.064 based on the results of Zavorsky et al. (2007) we estimated that ≥12 participants were needed to detect a mean difference of 30-sec in our primary end-point between SYN and CTN conditions, where 30-sec is equivalent to a worthwhile enhancement of 1.5% (Paton and Hopkins, 2005, 2006) in exercise performance assuming a mean time to completion of 33.5 min (Zavorsky et al., 2007).

We used two-tailed paired *t*-tests to compare the effects of SYN vs. CTN clothing ensembles on: 20kmCTT duration (primary endpoint); ΔBM; exercise-induced change in clothing ensemble mass; and sweat rate (SigmaStat; Systat Software Inc., San Jose, CA, USA). The effect of clothing ensemble (SYN vs. CTN), measurement time (Rest, 2-km, 4-km, …, 20-km) and their interaction on temperature, cardiac, metabolic, ventilatory, perceptual and cycle performance variables was examined using a two-tailed two-way repeated measures analysis of variance in combination with Tukey's HSD *post-hoc* test. Statistical significance was set at *p* < 0.05 and values are reported as mean ± SE.

## Results

### 20-Km Cycling Time Trial Performance

**Figure 2:**
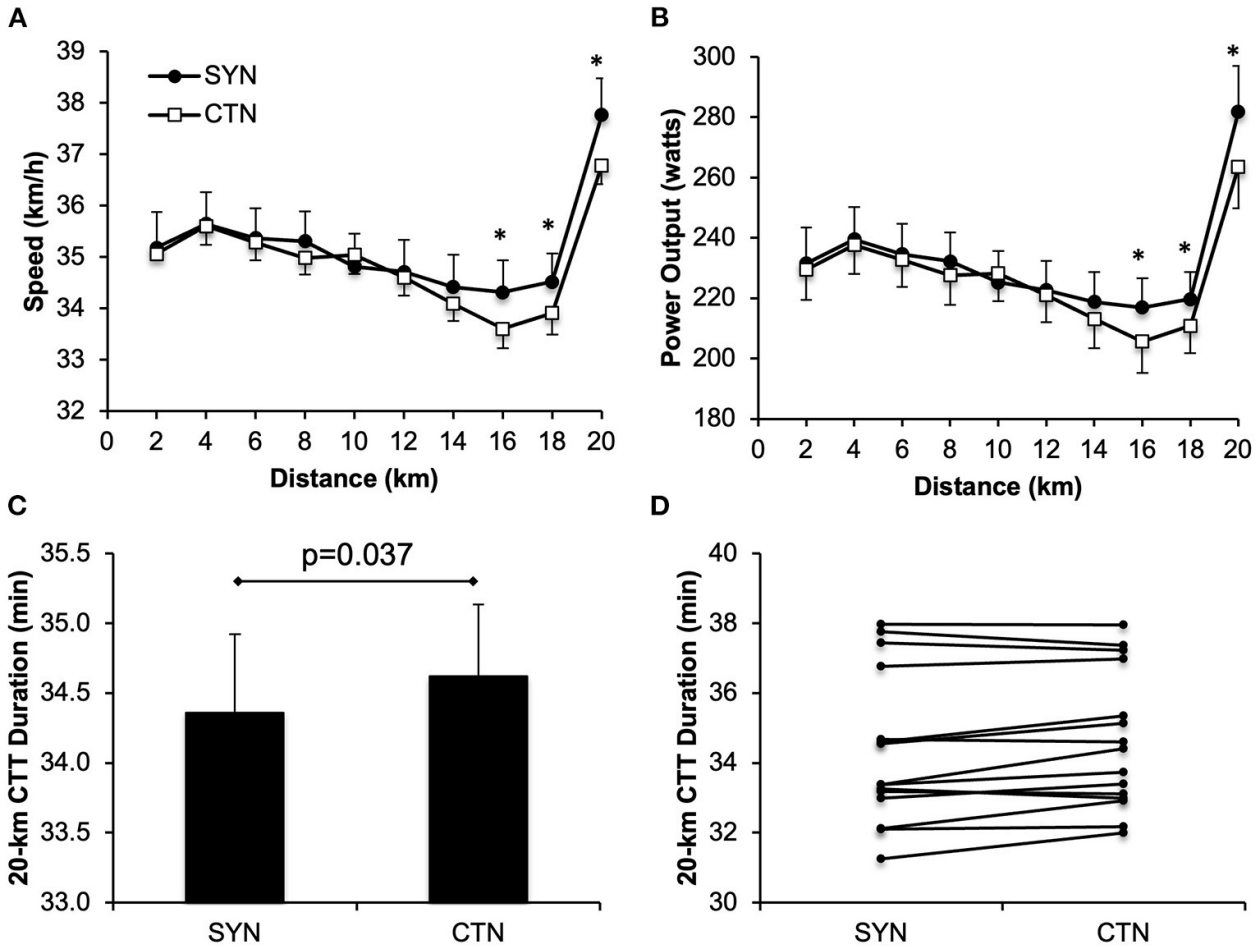
Effect of wearing athletic clothing made of synthetic fabrics (SYN, 60% polyester:40% nylon) and 100% cotton fabric (CTN) on 20-km cycling time trial (CTT) performance parameters in endurance-trained adults (*n* = 15). Values in panels **(A–C)** are means ± SE. Data points in panel **(D)** are individual participant values under SYN and CTN conditions. *p < 0.05 vs. CTN.

As illustrated in Figures 2A,B, participants maintained a significantly higher speed and power output over the last 6-km of the 20kmCTT while wearing the SYN vs. CTN ensemble: mean cycling speed was 0.71 km/hr (+2.0%), 0.61 km/hr (+1.8%) and 0.98 km/hr (+2.7%) faster at the 16, 18 and 20-km mark of the 20kmCTT, respectively; and mean power output was 11.3 watts (+5.5%), 8.8 watts (+4.2%) and 18.4 watts (+7.0%) higher at the 16, 18 and 20-km mark of the 20kmCTT, respectively. Hence, the time needed to complete the 20kmCTT was significantly reduced by 15.7 ± 6.8 sec under SYN vs. CTN conditions, representing a 0.8 ± 0.3% improvement in 20kmCTT performance with SYN. A beneficial effect of SYN vs. CTN (i.e., reduced 20kmCTT duration) was observed in 9/15 (60%) of the participants. 1/15 (7%) participant had similar 20kmCTT duration, and 5 had longer duration for the SYN compared to CTN (Figures 2C,D). To rule out a potentially confounding order effect on exercise performance, we compared 20kmCTT duration between *Visits 2* and *3* (i.e., independent of clothing ensemble) and found no statistically significant difference: 34.40 ± 0.55 vs. 34.59 ± 0.53 min (*p* = 0.144 by two-tailed paired *t*-test).

### Thermoregulatory Responses

Whole body sweat rate was not different between SYN vs. CTN conditions: 1.18 ± 0.17 vs. 1.26 ± 0.13 L/h, respectively (*p* = 0.160). The percentage change in body mass loss from rest to the end of the 20kmCTT tended to be lower under SYN vs. CTN conditions: −0.91 ± 0.09% vs. −0.98 ± 0.07%, respectively (*p* = 0.053). Exercise-induced increases in clothing mass were lower under SYN vs. CTN conditions: 50.5 ± 16.9 g vs. 76.0 ± 22.0 g, respectively (*p* = 0.001).

**Figure 3:**
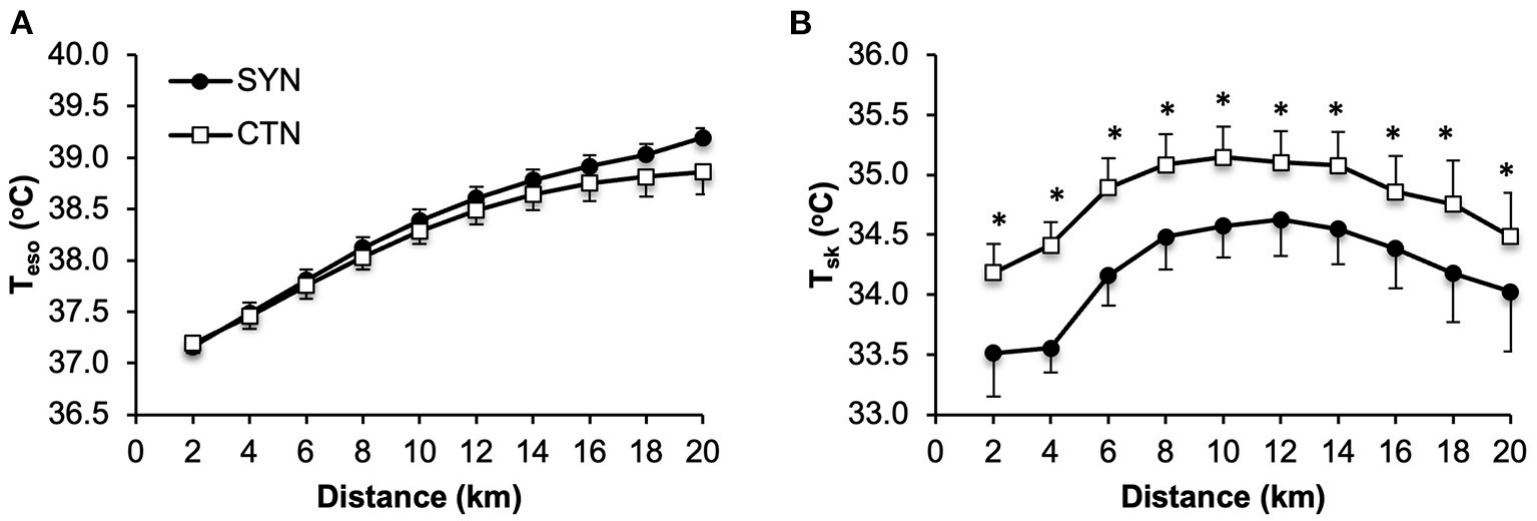
Effect of wearing athletic clothing made of synthetic fabrics (SYN, 60% polyester: 40% nylon) and 100% cotton fabric (CTN) on **(A)** esophageal temperature (Teso) and **(B)** whole body mean skin temperature ($\bar{T}sk$) during a 20-km cycling time trial in endurance-trained adults (*n* = 15). Values are means ± SE. *p < 0.05 vs. CTN.

At rest, $\bar{T}sk$ was 0.60 °C lower under SYN vs. CTN clothing conditions (*p* = 0.002), while no differences in *T*_eso_ were observed between trials (Table 4). As shown in Figure 3A, no statistically significant differences in *T*_eso_ were observed at any time during the 20kmCTT performed while wearing the SYN vs. CTN ensemble. By contrast, $\bar{T}sk$ was significantly lower (by 0.50-0.90°C) for the duration of the 20kmCTT under SYN vs. CTN conditions Figure 3B.

**Table 4:** Baseline values of thermoregulatory, cardiometabolic, ventilatory, and perceptual parameters.

	**SYN**	**CTN**
*T*_eso_ [°C]	36.6 ± 0.1	36.6 ± 0.11
${\bar{T}}_{\text{sk}}$ [°C]	33.9 ± 0.4*	34.6 ± 0.2
VO_2_ [ml/kg/min]	6.6 ± 0.4	6.9 ± 0.3
Heart rate [bpm]	70 ± 2	73 ± 3
Ventilation [L/min]	13.6 ± 1.0	14.5 ± 1.0
RPE [0–10]	0 ± 0	0 ± 0
Clothing comfort [+3,−3]	0.7 ± 0.3	0.4 ± 0.3
Thermal sensation [+4,−4]	0.1 ± 0.2	−0.3 ± 0.2
Skin wettedness [3,−3]	0.7 ± 0.3	0.7 ± 0.3

Values are means ± SE [range]. T_es_, esophageal temperature; ${\bar{T}}_{\text{sk}}$, whole body mean skin; VO_2_, rate of oxygen uptake; RPE, rating of perceived exertion. *denotes p <0.05.

### Cardiometabolic and Ventilatory Responses

**Figure 4:**
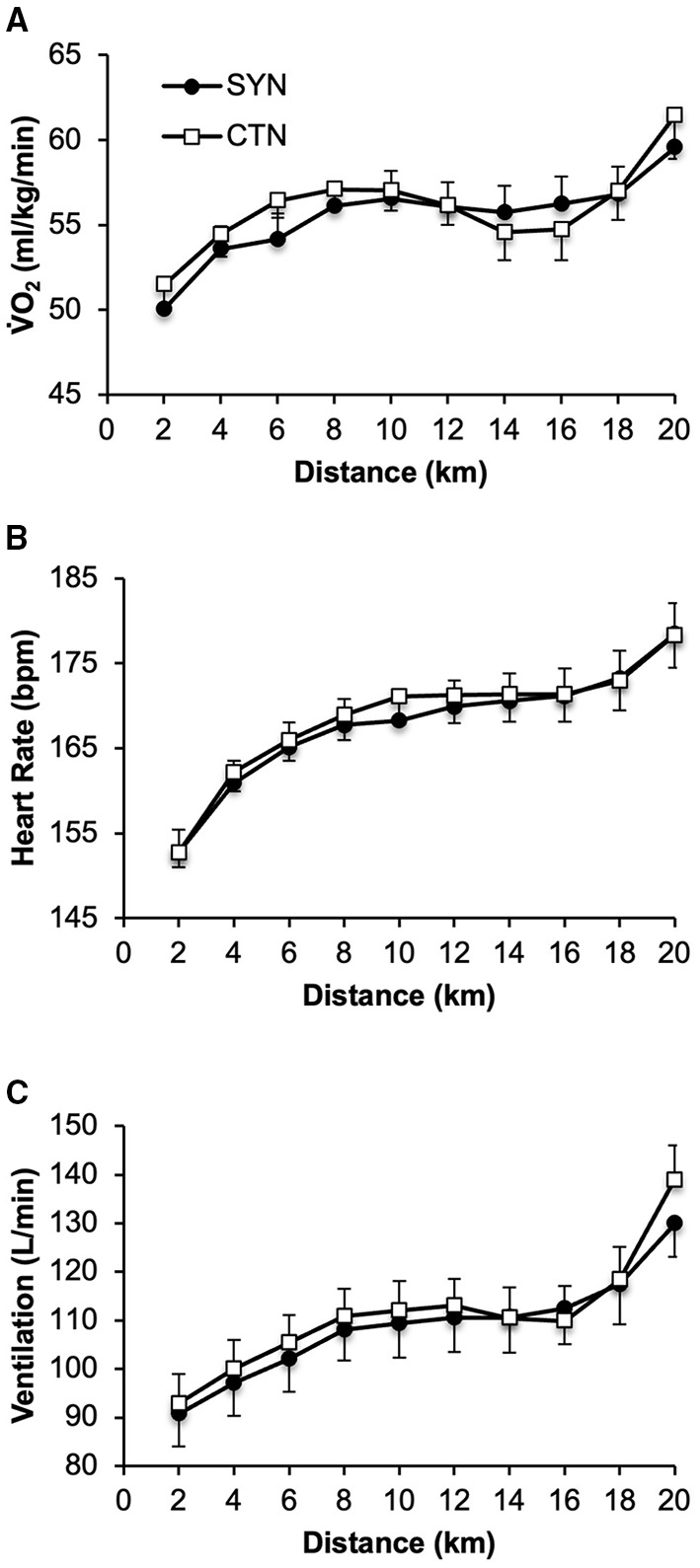
Effect of wearing athletic clothing made of synthetic fabrics (SYN, 60% polyester: 40% nylon) and 100% cotton fabric (CTN) on **(A)** metabolic, **(B)** cardiac and **(C)** ventilation responses during a 20-km cycling time trial in endurance-trained adults (*n* = 15). Values are means ± SE. VO_2_, rate of oxygen consumption.

Cardiac, metabolic and ventilatory responses were similar at rest (Table 4) and during the 20kmCTT under SYN vs. CTN clothing conditions (Figure 4).

### Perceptual Responses

**Figure 5:**
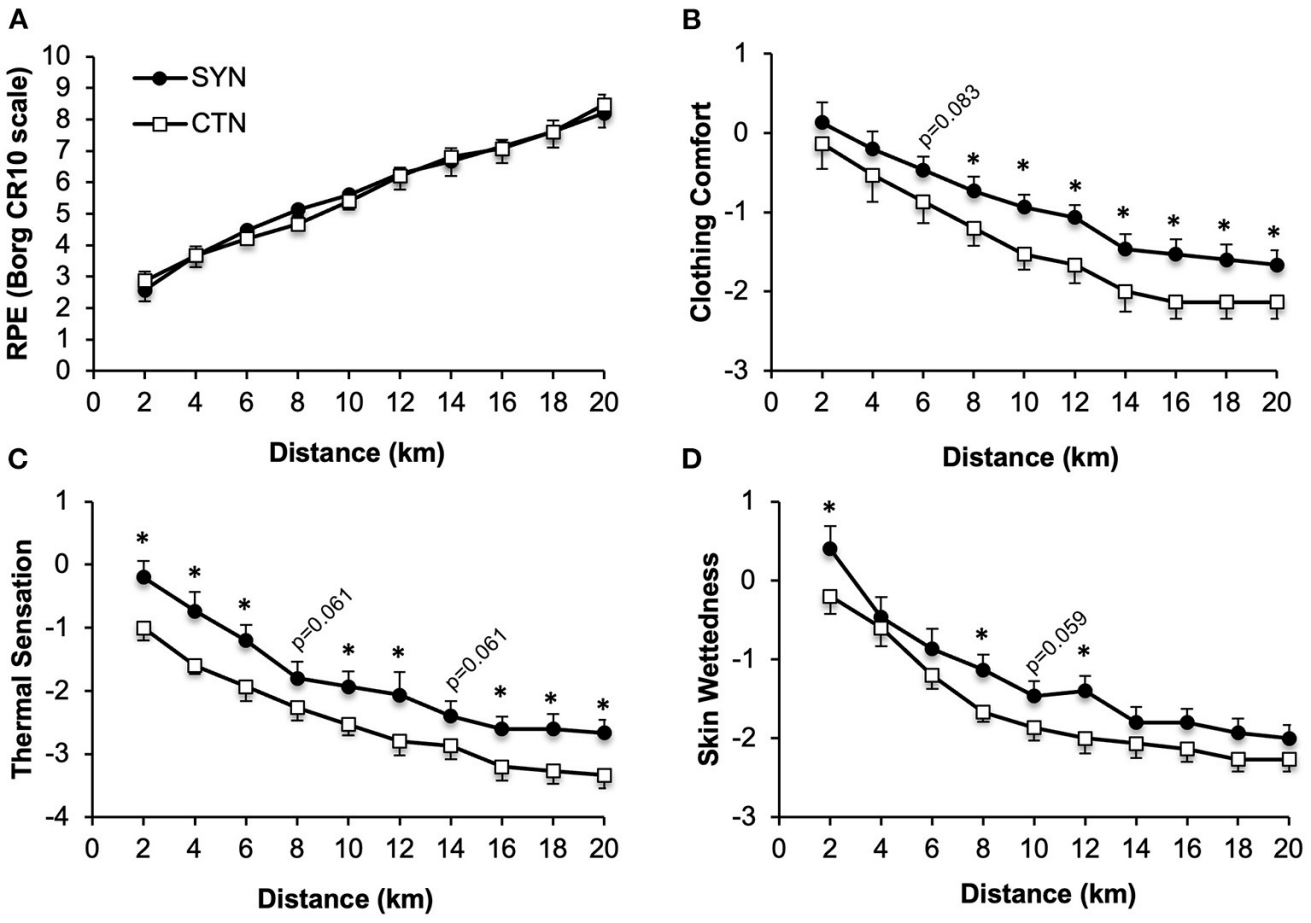
Effect of wearing athletic clothing made of synthetic fabrics (SYN, 60% polyester: 40% nylon) and 100% cotton fabric (CTN) on perceptual responses during a 20-km cycling time trial in endurance-trained adults (*n* = 15). Values are means ± SE. RPE, **(A)** rating of perceived exertion; CR10, 0-10 category ratio. **(B)** Participants rated clothing comfort using a 7-point scale (+3, very comfortable; +2, comfortable; +1, slightly comfortable; 0, neutral; −1, slightly uncomfortable; −2, uncomfortable; −3, very uncomfortable); **(C)** thermal sensation using a 9-point scale (−4, very hot; −3, hot; −2, warm; −1, slightly warm; 0, neutral; +1, slightly cool; +2, cool; +3, cold; +4, very cold); **(D)** skin wettedness using a 7-point scale (−3, too wet; −2, wet; −1, slightly wet; 0, neutral; +1, slightly dry; +2, dry; +3, too dry). *p < 0.05 vs. CTN.

Participant's ratings of perceived exertion, clothing comfort, thermal sensation and skin wettedness were not significantly different at rest while wearing SYN vs. CTN ensembles (Table 4). As shown in Figure 5A, ratings of perceived exertion during the 20kmCTT were virtually identical under SYN vs. CTN conditions. By contrast, participant's ratings of clothing comfort, thermal sensation and skin wettedness were consistently higher (or more neutral) while wearing the SYN vs. CTN clothing ensemble (Figures 5B–D).

### Debriefing Responses

Fourteen of 15 participants (93%) correctly identified the experimental visit that they were randomly assigned to wear the SYN clothing ensemble. Twelve of 15 participants (80%) felt that their performance was better while wearing the SYN clothing ensemble, with one participant (7%) reporting better performance with CTN and 2 participants (13%) unable to distinguish. Ten of the 12 (83%) participants who felt that they performed better while wearing the SYN clothing ensemble identified the clothing garment as a factor contributing to their perception of better performance, citing “*lighter,” “cooler,” “thinner,” “drier,” “more comfortable”* and “*more moisture wicking”* fabric as the reason(s). Fourteen of 15 participants (93%) felt that they experienced the least amount of thermal strain during the 20kmCTT test performed while wearing the SYN clothing ensemble; and that the SYN clothing ensemble provided the best “cooling” effect during the 20kmCTT.

## Discussion

The main findings of this randomized, controlled, crossover study are as follows: (1) 20kmCTT duration was significantly reduced by an average of 15.7 sec or 0.8% under SYN vs. CTN clothing conditions, reflecting improved exercise performance and consequent to maintenance of a significantly faster speed and higher power output over the last 6-km of the 20kmCTT; and (2) improved 20kmCTT performance under SYN vs. CTN clothing conditions was associated with maintenance of a significantly lower skin temperature and more favorable ratings of thermal sensation and clothing comfort throughout exercise.

### Effect of Clothing Fabric on Exercise Performance

The smallest valuable change in performance time affecting an elite cyclist or triathlete's chances of winning or medaling in a competition is ~0.5% (Zavorsky et al., 2007; Faul et al., 2009). Indeed, the performance times separating the Gold and Silver medalists, the Silver and Bronze medalists, and the Bronze medalist and each of the 4^th^ and 5^th^ place finishers of the Men's 60-km road cycling time trial at the Rio 2016 Olympic Games were 47 sec (1.08%), 15 sec (0.34%), 4 sec (0.09%) and 8 sec (0.18%), respectively. Notwithstanding obvious differences in technical demands and environmental conditions between laboratory-based (simulated) and actual cycling time trials, (Brown and Banister, 1985) the results of the current study, demonstrating 0.8% improved performance time, are among the first to demonstrate that clothing fabric has a statistically significant and potentially meaningful effect on human exercise performance.

### Mechanisms of Improved Exercise Performance

Thermoregulatory behaviors during self-paced exercise have been suggested to be mediated by changes in ratings of perceived exertion and subjective indices of thermal comfort and sensation, with alterations in skin temperature playing a critical role in directing thermo-behavioral adjustments in exercise intensity (Schlader et al., 2010, 2011a,b,c). Furthermore, in a recent study, it was found that *T*_skin_, but not internal/core temperature, was a strong predictor of physical work capacity (Foster et al., 2021). Thus, maintenance of a lower *T*_skin_ and the accompanying optimization of perceived thermal and clothing sensations throughout exercise under SYN vs. CTN conditions may have facilitated a thermo-behavioral increase in 20kmCTT performance without simultaneously increasing ratings of perceived exertion. During the last 6-km of the 20kmCTT, the participants maintained higher speeds and power outputs with better (more neutral) perceived thermal sensations and clothing comfort. This higher power output was reflected in *T*_eso_ values, that trended toward being higher in the SYN vs. CTN condition, suggesting that perception of *T*_skin_ drives self-selected exercise intensity independent of core temperature, and further support the previous reports (Schlader et al., 2010, 2011a,b,c; Foster et al., 2021).

The relatively constant difference in Tskin between the SYN and CTN conditions observed throughout the baseline and exercise periods (pre and post the onset of sweat), may be attributed to the lower thermal resistance (~3 fold) and higher air permeability (~3 fold) measured for the SYN vs. CTN, that facilitated enhanced heat dissipation via conduction and convection. The vapor resistance, which was ~3 fold lower for SYN vs. CTN, further enabled greater evaporative heat loss (or mitigated the interference to evaporative heat loss) in the SYN ensemble. We estimate that under the ambient conditions and clothing configurations tested in this study, moisture management (wicking) had less contribution to the beneficial Tskin profile and the subsequent improved performance.

Despite CTN demonstrating superior stretchability compared to SYN, the SYN fabric's greater breathability, lower insulation, lower water vapor resistance and lower recovery were significant and distinguishable from CTN, as almost all of the participants defined the SYN as the “cooler” clothing ensemble, and their ratings of thermal sensation and clothing comfort were consistently better for the SYN compared to CTN.

Our results reinforce those of Jiao et al., who found that optimized clothing with higher heat dissipation capacity due to their body-mapping design and advanced fabrics resulted in a large decrease in skin temperature and small increase in core temperature of participants at rest and during constant running exercise compared to non-optimized synthetic clothing (Jiao and Yao, 2017). Furthermore, the optimized clothing improved participants' subsequent all-out running performance by 8% after constant running and decrease the risk of heat stress during running in a hot environment (Jiao and Yao, 2017).

### Methodological Considerations

The primary aim of this study was to examine the effect of SYN vs. CTN clothing ensembles on exercise performance and its thermoregulatory, cardiometabolic and perceptual determinants in endurance-trained cyclists and triathletes. We employed a 20kmCTT, which better represents the dynamic power management pattern that is expected during athletic competition compared to the constant power output cycle and treadmill tests used in most previous studies, in which no consistent effect of clothing fabric on thermoregulatory and perceptual responses to exercise have been reported (Kwon et al., 1998; Brazaitis et al., 2010; De Sousa et al., 2014; Abdallah et al., 2015). The loose-fitting long-sleeved shirts and fitted full-length trousers maximized the likelihood of demonstrating an effect of SYN vs. CTN fabrics on measured variables by minimizing the nude skin surface area for evaporative heat loss. It is reasonable to suggest that earlier studies failed to demonstrate an effect of synthetic vs. natural fabrics on thermoregulatory and perceptual responses to exercise because, unlike us, they used semi-nude clothing ensembles (i.e., shorts with a long or short-sleeved shirt) that permitted heat loss independent of the fabrics under investigation. Another possible source for the lack of effect of fabric on thermoregulation could be due to similar fabric moisture management capacity of the selected garments (Gavin et al., 2001; Brazaitis et al., 2010; Sperlich et al., 2013; De Sousa et al., 2014).

Even though SYN and CTN clothing ensembles were of comparable density and identical bi-layer snowflake mesh construction, it is clear from the debriefing responses that it was not possible to blind our participants to the randomly assigned test fabrics due to obvious differences in their texture. Unfortunately, the extent to which participant ‘unblinding' contributed to the observed differences in 20kmCTT performance, and subjective ratings of thermal sensation and clothing comfort during exercise under SYN vs. CTN clothing conditions cannot be determined from our study; thus, we cannot rule out a potentially confounding placebo effect.

Our study was carried out under ambient laboratory conditions of 24.3 ± 0.7°C and 17 ± 7% relative humidity with a simulated wind of ~3 m/s, which can be considered as mild environmental heat load and favorable for heat dissipation by evaporation. Nevertheless, the high metabolic demand of exercise (VO_2_ ≥ 50 ml/kg/min) was sufficient to increase *T*_eso_ by ~2°C from rest under these mild ambient conditions. We speculate that the observed effects of SYN vs. CTN fabrics on 20kmCTT duration, $\bar{T}sk$, and ratings of perceived thermal sensation and clothing comfort may have been further exaggerated in the setting of longer activities in which sweat accumulates even further. Additional research is needed to assess whether our results can be replicated under an uncompensable hot (≥30°C) and humid (≥60%) environment with higher simulated wind speeds that better mimic the outdoor conditions of an actual athletic competition.

Finally, most cyclists and triathletes would not train and/or compete in full-length trousers and a long-sleeved shirt. Thus, we must concede that the results of our laboratory-based study may not be generalizable to the outdoor conditions of endurance athletes. However, our study has implications for military personnel, firefighters, and first responders, who are often required to wear full body clothing ensembles and perform high intensity physical activity in hot and/or humid environments. In these populations, the prevention of heat-related performance decrements, and potentially lethal exertional heat illness, are of high importance; thus, even a slight improvement in the thermal properties of a clothing ensemble (as observed in our study) may be beneficial (Taylor and Patterson, 2014).

## Practical Applications

The current study suggests that athletic performance (speed and power) can be improved by maintaining lower *T*_skin_, through using more breathable clothing (with lower vapor and thermal resistance).

## Conclusion

Under the experimental conditions of the current study, we conclude that athletic clothing made of synthetic fabrics with lower insulation and evaporative resistance compared to similar clothing ensemble made of 100% cotton fabric, significantly improved laboratory-based 20-km cycling time trial performance by decreasing Tskin and optimizing selected thermoregulatory and perceptual responses to exercise in endurance-trained cyclists and triathletes.

## Data Availability Statement

The raw data supporting the conclusions of this article will be made available by the authors, without undue reservation.

## Ethics Statement

The studies involving human participants were reviewed and approved by Institutional Review Board of the Faculty of Medicine at McGill University (A01-M12-15BB). The patients/participants provided their written informed consent to participate in this study.

## Author Contributions

JF and DJ: concept, design, collection, assembly, and analysis of data. JF, AH, YE, and DJ: interpretation of results and drafting the article and/or revising it critically for important intellectual content. All authors approved the final version of the manuscript, while DJ is accountable for all aspects of the work.

## Funding

Operating funds for this work were provided by an unrestricted and investigator-initiated grant from Lamour Hosiery Inc. (Montréal, QC, Canada). AH was supported by a McGill Sports Science Post-Doctoral Research Fellowship. DJ was supported by a Chercheurs-Boursiers Junior 1 salary award from the Fonds de Recherche du Québec-Santé and by a William Dawson Research Scholars Award (McGill University). DJ holds a Canada Research Chair, Tier II, in Clinical Exercise and Respiratory Physiology from the Canadian Institutes of Health Research. The financial sponsor, Lamour Hosiery Inc. (Montréal, QC, Canada), had no role in the conduct of the study, data analysis and conclusions, manuscript preparation, or manuscript review.

## Conflict of Interest

The authors declare that the research was conducted in the absence of any commercial or financial relationships that could be construed as a potential conflict of interest.

## Publisher's Note

All claims expressed in this article are solely those of the authors and do not necessarily represent those of their affiliated organizations, or those of the publisher, the editors and the reviewers. Any product that may be evaluated in this article, or claim that may be made by its manufacturer, is not guaranteed or endorsed by the publisher.

## References

[B1] Abdallah S. J. Krug R. Jensen D. (2015). Does wearing clothing made of a synthetic “cooling” fabric improve indoor cycle exercise endurance in trained athletes? Physiol. Reports. 3, e12505. 10.14814/phy2.12505 26290527 PMC4562588

[B2] Borg G. A.. (1982). Psychophysical bases of perceived exertion. Med. Sci. Sports Exerc. 14, 377–381. 10.1249/00005768-198205000-00012 7154893

[B3] Brazaitis M. Kamandulis S. Skurvydas A. Daniuseviciute L. (2010). The effect of two kinds of T-shirts on physiological and psychological thermal responses during exercise and recovery. Comparative Study. Appl. Ergonom. 42, 46–51. 10.1016/j.apergo.2010.04.001 20427033

[B4] Brown S. L. Banister E. W. (1985). Thermoregulation during prolonged actual and laboratory-simulated bicycling. Eur. J. Appl. Physiol. Occupat. Physiol. 54, 125–130. 10.1007/BF00426312 4018048

[B5] Corbett J. Barwood M. J. Tipton M. J. (2015). Physiological cost and thermal envelope: a novel approach to cycle garment evaluation during a representative protocol. Scandinav. J. Med. Sci. Sports. 25, 152–158. 10.1111/sms.12176 24433540

[B6] Davis J. K. Bishop P. A. (2013). Impact of clothing on exercise in the heat. Sports Med. 43, 695–706. 10.1007/s40279-013-0047-8 23620245

[B7] De Sousa J. Cheatham C. Wittbrodt M. (2014). The effects of a moisture-wicking fabric shirt on the physiological and perceptual responses during acute exercise in the heat. Appl. Ergonom. 45, 1447–1453. 10.1016/j.apergo.2014.04.006 24768089

[B8] Faul F. Erdfelder E. Buchner A. Lang A. G. (2009). Statistical power analyses using G^*^Power 3.1: tests for correlation and regression analyses. Behav. Res. Methods. 41, 1149–1160. 10.3758/BRM.41.4.1149 19897823

[B9] Foster J. Smallcombe J. W. Hodder S. Jay O. Flouris A. D. Nybo L. (2021). An advanced empirical model for quantifying the impact of heat and climate change on human physical work capacity. Int. J. Biometeorol. 65, 1215–1229. 10.1007/s00484-021-02105-0 33674931 PMC8213606

[B10] Gavin T. P.. (2003). Clothing and thermoregulation during exercise. In: *Research Support, Non-U.S. Gov't Research Support, U.S. Gov't, P.H.S. Sports Med*. 33, 941–947. 10.2165/00007256-200333130-00001 14606923

[B11] Gavin T. P. Babington J. P. Harms C. A. Ardelt M. E. Tanner D. A. Stager J. M. (2001). Clothing fabric does not affect thermoregulation during exercise in moderate heat. Med. Sci. Sports Exerc. 33, 2124–2130. 10.1097/00005768-200112000-00023 11740309

[B12] Gonzalez-Alonso J. Teller C. Andersen S. L. Jensen F. B. Hyldig T. Nielsen B. (1999). Influence of body temperature on the development of fatigue during prolonged exercise in the heat. J. Appl. Physiol. 86, 1032–1039. 10.1152/jappl.1999.86.3.1032 10066720

[B13] Hooper D. R. Cook B. M. Comstock B. A. (2015). Synthetic garments enhance comfort, thermoregulatory response, and athletic performance compared with traditional cotton garments. J. Strength Condit. Res. 29, 700–707. 10.1519/JSC.0000000000000783 25463694

[B14] Jiao J. Yao L. Y. (2017). Effects of body-mapping-designed clothing on heat stress and running performance in a hot environment. Ergonomics. 60, 1435–1444. 10.1080/00140139.2017.1306630 28306388

[B15] Kenny G. P. Jay O. (2013). Thermometry, calorimetry, and mean body temperature during heat stress. Comprehens. Physiol. 3, 1689–1719. 10.1002/cphy.c130011 24265242

[B16] Kwon A. Kato M. Kawamura H. Yanai Y. Tokura H. (1998). Physiological significance of hydrophilic and hydrophobic textile materials during intermittent exercise in humans under the influence of warm ambient temperature with and without wind. Eur. J. Appl. Physiol Occupat. Physiol. 78, 487–493. 10.1007/s004210050450 9840402

[B17] Park C. R. Palmes E. D. (1947). Thermal regulation during early acclimatization to work in a hot dry environment.27900769

[B18] Paton C. D. Hopkins W. G. (2005). Competitive performance of elite olympic-distance triathletes: reliability and smallest worthwhile enhancement. Sportscience. 9, 1–5. Available online at: 10.1080/17461390500422796

[B19] Paton C. D. Hopkins W. G. (2006). Variation in performance of elite cyclists from race to race. Eur. J. Sport. Sci. 6, 25–31. 10.1080/17461390500422796

[B20] Plowman S. Smith D. (2011). Exercise Physiology for Health, Fitness and Performance. Lippincott Williams and Wilkins.

[B21] Rowell L. B. Marx H. J. Bruce R. A. Conn R. D. Kusumi F. (1966). Reductions in cardiac output, central blood volume, and stroke volume with thermal stress in normal men during exercise. J. Clin. Investigat. 45, 1801–1816. 10.1172/JCI105484 5926447 PMC292862

[B22] Sawka M. N. Cheuvront S. N. Kenefick R. W. (2012). High skin temperature and hypohydration impair aerobic performance. Experiment. Physiol. 97, 327–332. 10.1113/expphysiol.2011.061002 22143882

[B23] Sawka M. N. Wenger C. (1988). Physiological Responses to Acute Exercise-Heat stress.

[B24] Schlader Z. J. Simmons S. E. Stannard S. R. Mundel T. (2011a). The independent roles of temperature and thermal perception in the control of human thermoregulatory behavior. Physiol. Behav. 103, 217–224. 10.1016/j.physbeh.2011.02.002 21315099

[B25] Schlader Z. J. Simmons S. E. Stannard S. R. Mundel T. (2011b). Skin temperature as a thermal controller of exercise intensity. Eur. J. Appl. Physiol. 111, 1631–1639. 10.1007/s00421-010-1791-1 21197543

[B26] Schlader Z. J. Stannard S. R. Mundel T. (2010). Human thermoregulatory behavior during rest and exercise - a prospective review. Physiol. Behav. 3 99, 269–275. 10.1016/j.physbeh.2009.12.003 20006632

[B27] Schlader Z. J. Stannard S. R. Mundel T. (2011c). Evidence for thermoregulatory behavior during self-paced exercise in the heat. J. Thermal. Biol. 36, 390–396. 10.1016/j.jtherbio.2011.07.00221315099

[B28] Sperlich B. Born D. P. Lefter M. D. Holmberg H. C. (2013). Exercising in a hot environment: which T-shirt to wear? Wilderness Environ Med. 24, 211–220. 10.1016/j.wem.2013.04.005 23870763

[B29] Steudel-Numbers K. L.. (2003). The energetic cost of locomotion: humans and primates compared to generalized endotherms. J. Human Evol. 44, 255–262. 10.1016/S0047-2484(02)00209-9 12662945

[B30] Taylor N. A. Patterson M. J. (2014). Military clothing and protective material: protection at the limits of physiological regulation. The Mechanobiology and Mechanophysiology of Military-Related, Injuries. Springer. p. 303–332. 10.1007/8415_2014_181

[B31] Wang F. Annaheim S. Morrissey M. Rossi R. (2014). Real evaporative cooling efficiency of one-layer tight-fitting sportswear in a hot environment. Scandinav. J. Med. Sci. Sports. 24, e129–e139. 10.1111/sms.12117 24033668

[B32] Wang F. Cai X. Zhang C. Shi W. Lu Y. Song G. (2016). Assessing the performance of a conceptual tight-fitting body mapping sportswear (BMS) kit in a warm dry environment. Fiber. Polym. 17, 151–159. 10.1007/s12221-016-5375-5

[B33] Zavorsky G. S. Murias J. M. Gow J. (2007). Laboratory 20-km cycle time trial reproducibility. Comparative Study. Int. J. Sport Med. 28, 743–748. 10.1055/s-2007-964969 17455116

